# Monoarthritis of the knee revealing tabetic arthropathy

**DOI:** 10.11604/pamj.2017.26.100.11683

**Published:** 2017-02-24

**Authors:** Zeineb Alaya, Walid Osman

**Affiliations:** 1 Department of Rheumatology, Farhat Hached Hospital, Faculty of Medicine of Sousse, Sousse, Tunisia; 2 Department of Orthopaedics, Sahloul Hospital, Faculty of Medicine of Sousse, Sousse, Tunisia

**Keywords:** Monoarthritis, knee, syphilis

## Image in medicine

53-year-old man presented with monoarthritis of the knee without fever evolving since 6 months associated with an inflammatory biological syndrome. The articular puncture brought back a sterile inflammatory fluid. The radiographs of the frontal knee (A) and profile knee (B) and CT of the knee (C, D) showed destruction of the internal femoral condyle with osteolysis of the medial border of the internal tibial plateau associated with multiple bone constructions with the presence of intra-articular fragments, intra-articular effusion and thickening of the synovium. The diagnosis of tabetic arthropathy in its hypertrophic form was retained following a 20-year history of syphilitic inoculation chancre, posterior radiculocordonal syndrome, imaging data and positive syphilitic serology (TPHA-VDRL) in the blood and cerebrospinal fluid. The patient was treated with penicillin G (24 million/day) for 15 days. Tabetic arthropathy is a type of neuropathic arthropathy that has become rare and unfamiliarity with the clinical presentation of this disease may lead to considerable delay in diagnosis. This disease means progressive painless joint destruction that is related to neurosensory deficits caused by syphilis. His diagnosis is difficult as its clinical presentation is not specific and differential diagnosis is wide ranging. Hence, his diagnosis requires clinical suspicion and an appropriate serological test.

**Figure 1:**
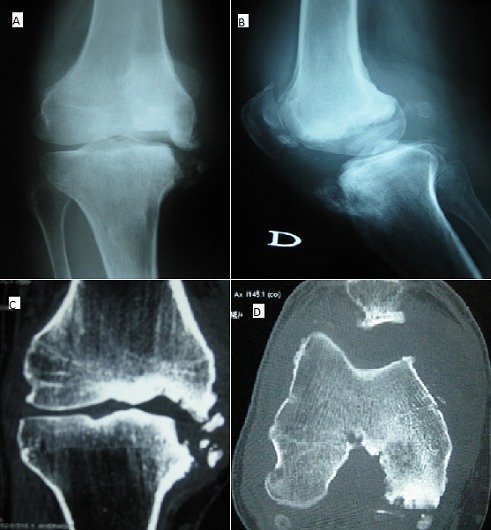
(A, B) radiography of the right knee (face and profile incidence): destruction of the internal femoral condyle with osteolysis of the internal tibial plateau associated with multiple bone constructions; (C, D) knee scan in sagittal and axial section: destruction of the internal femoral condyle with osteolysis of the medial border of the internal tibial plateau associated with multiple bone constructions with the presence of intra-articular fragments, intra-articular effusion and thickening of the synovium

